# Surface expression of CXCR4 on circulating CD133^+ ^progenitor cells is associated with plaque instability in subjects with carotid artery stenosis

**DOI:** 10.1186/2040-2384-1-10

**Published:** 2009-12-27

**Authors:** Dominik Sepp, Lorena Esposito, Peter Zepper, Ilka Ott, Regina Feurer, Suwad Sadikovic, Bernhard Hemmer, Holger Poppert

**Affiliations:** 1 Department of Neurology, Klinikum rechts der Isar, Technische Universitaet Muenchen, Germany; 2 Department of Cardiology, Klinikum rechts der Isar, Technische Universitaet Muenchen, Germany

## Abstract

**Background:**

Circulating progenitor cells (PCs) are considered to contribute to the remodeling of atherosclerotic plaques. Their surface receptor CXCR4 plays an important role in the recruitment of PCs to their target. This study compares the mobilization of PCs and their functional characteristics in asymptomatic subjects with stable and with unstable carotid plaques. This could provide insight into plaque remodeling and help to develop biomarkers for plaque stability.

**Methods:**

In 31 subjects with asymptomatic carotid artery stenosis we analyzed the number of CD133^+ ^PCs, VEGFR2^+^CD34^+ ^PCs and the surface expression of CXCR4 on CD133^+ ^PCs by flow cytometry. Subjects underwent bilateral carotid MRI in a 1.5-T scanner in order to allow the categorization of plaques, following the modified criteria of the American Heart Association.

**Results:**

The number of CD133^+ ^PCs and VEGFR2^+^CD34^+ ^PCs showed no significant difference between subjects with stable and unstable carotid plaques. The expression of CXCR4 on CD133^+ ^PCs was higher in subjects with unstable plaques than in subjects with stable plaques (p = 0.009).

**Conclusions:**

This study demonstrates an association between functional characteristics of circulating CD133^+ ^PCs and plaque stability in subjects with asymptomatic carotid artery stenosis. The higher expression of CXCR4 on CD133^+ ^PCs suggests a difference in the recruitment of PCs to the injured tissue in subjects with unstable plaques and subjects with stable plaques. As surface expression of CXCR4 on CD133^+ ^PCs differs in subjects with unstable and with stable plaques, CXCR4 is a promising candidate for a serological biomarker for plaque stability.

## Background

Atherosclerotic carotid artery stenosis represents a leading risk factor for ischemic stroke. The degree of vessel stenosis still represents the main parameter for determining atherosclerotic disease severity. However, the individual stroke risk of patients presenting with asymptomatic severe carotid artery stenosis is relatively low considering that around 11% of these patients experience an ischemic stroke over 5 years [1]. These data suggest that a risk stratification based solely on luminal patency fails to reflect our current understanding of the pathophysiology of atherosclerosis. Histological studies suggest that plaque composition is more important than plaque burden: carotid lesions with intraplaque hemorrhage or a lipid-rich necrotic core are regarded as unstable, high-risk plaques [2-4]. In contrast, plaque lesions consisting mainly of fibrous tissue or containing large amounts of calcification are regarded as stable plaques [5,6]. High-resolution magnetic resonance imaging (MRI) represents a new technique with the ability to visualize such plaques non-invasively [7-9]. Based on histological American Heart Association (AHA) criteria, modified specifically for MRI use, a classification was introduced by Cai and co-workers that allows categorization of carotid plaque features noninvasively into distinct lesion types (I-VIII) [4,10,11]. According to this modified classification, plaque lesions characterized by a lipid-rich necrotic core, by the presence of a fibrous cap or by intraplaque hemorrhage represent lesion types IV-VI and are regarded as high-risk, unstable plaques that are likely to rupture and lead to cerebral ischemia [2,3,5,6,12-16]. Previous literature suggests that inflammation may represent an important factor in the progression of atherosclerotic lesions [17,18]. Yet the precise underlying mechanisms of atherosclerosis and plaque formation are still not fully understood.

Circulating progenitor cells (PCs), which are derived from adult stem cells, are known to contribute to vascular repair and may play a pivotal role in the development of atherosclerotic plaques. Two functions of PCs are of interest in this context. They are involved in reendothelialization after endothelial damage, which could be beneficial by decreasing the development of atherosclerotic lesions [19]. By contrast, their contribution to neovascularization could promote the progression of atherosclerosis by enhancing the entry of inflammatory cells and cytokines into the arterial wall [20-23].

The number of circulating PCs is reduced in patients with cardiovascular risk factors by decreased mobilization from the bone marrow and increased recruitment of PCs to sites of vascular injury [24,25]. By contrast, mobilization of PCs is increased during an ischemic event like myocardial ischemia and limb ischemia [23,26]. Statins, which are considered as plaque-stabilizing, increase the number of PCs in patients with coronary artery disease [27].

Not only the mobilization of PCs into the blood circulation but also the recruitment to their target, the "homing", must be considered when observing the function of progenitor cells. CXCR4, a chemokine receptor, that is expressed on PCs has been reported to play an important role for the homing of PCs [28]. It interacts with stromal cell-derived factor 1 (SDF-1), which is expressed by endothelial cells and fibroblasts and is increased during tissue damage such as in myocardial ischemia [29,30]. By analyzing the expression of CXCR4 on PCs it is possible to study a functional parameter of PCs.

While circulating PCs seem to be reduced in the blood of patients with carotid plaques, they might still play a role for the plaque remodeling [31].

The identification of distinct plaque components is important in correlating PCs with plaque stability. As plaque imaging by MRI is a reliable and noninvasive method for distinguishing stable and unstable plaques, it can be used to determine the stability of a plaque without the need to obtain a histological sample [9,32,33].

The aim of our study was to analyze the relationship of circulating PCs and their CXCR4 expression with plaque stability in order to provide insight into the plaque remodeling and thus find a potential marker for the risk stratification of plaques.

## Methods

### Subject Selection

We recruited 31 consecutive patients at our neurovascular clinic with carotid artery stenosis. Subjects were included who presented a carotid stenosis >50% diagnosed by duplex sonography (ECST criteria). Subjects having a history of TIA or stroke within the previous 6 months were excluded. Further exclusion criteria were anamnestic chronic inflammatory, malign, and chronic infectious diseases as well as cardial and peripheral limb ischemia within the previous 6 months. All subjects gave written informed consent before inclusion in the study. The examinations were performed between April and June 2007.

### Risk factor evaluation

Medical history, current medication and smoking habits were assessed. The clinical examination included a physical status, blood pressure measurement, blood tests, a 12-lead ECG and an ultrasound examination of the carotid arteries. Diabetes mellitus was defined as a fasting glucose level >126 mg/dl, glucose level at any time >200 mg/dl, use of hypoglycemic agents, or a history of physician-diagnosed diabetes mellitus. Hypertension was defined as systolic blood pressure >140 mmHg or diastolic blood pressure >90 mmHg in the supine position, or use of antihypertensive medication. Hyperlipidemia was defined as a fasting cholesterol value >240 mg/dl, low-density lipoprotein (LDL) cholesterol >190 mg/dl, LDL/high-density lipoprotein (HDL) ratio >4.0, or a history of physician-diagnosed increased cholesterol.

### MRI Protocol

All subjects were imaged with a 1.5-T scanner (Magnetom Symphony Quantum Gradient; Siemens Medical System; Germany) with bilateral phased-array surface coils (PACC-SS15; Machnet B.V., Netherlands). According to our previously published protocol, four contrast-weighted images were obtained as follows: 3-dimensional time-of-flight MR angiography (3D TOF), T1-weighted (T1w), T2-weighted (T2w), and proton-density-weighted (PDW) studies of both carotid arteries. The MRI scan was centered on the carotid bifurcation on the side of the stenosis to assure proper matching between the contrast-weighted imaging series of each patient. The imaging sequences were as follows: 3D TOF: field of view (FOV) 200 mm/75.0%; repetition time (TR) 43 ms; time to echo (TE) 7.15 ms, number of excitations (NEX) 2. T1w: FOV 160 mm/100%; TR 700 ms; TE 14 ms; NEX 2. T2w: FOV 160 mm/100%; TR 700 ms; TE 100 ms; NEX 2. PDW: FOV 160 mm/100%; TR 700 ms; TE 10 ms; NEX 2. Slice thickness was 1 mm for the 3D TOF and 2 mm for the T1w, T2w, and PDW images. The longitudinal coverage of each carotid artery was 72 mm (72 slices) for the 3D TOF and 24 mm (12 slices) for T1w, T2w, and PDW images.

The subjects were positioned on a vacuum pillow to avoid head-neck region movement during the MRI scan to ensure proper alignment between the images acquired in the four contrast-weighted imaging sequences of each patient.

### MR Image Review

Before evaluating the MRI scans, an image quality rating (4-point scale; 1 = best, 4 = worst) was assigned to all MR images for each contrast-weighted-image. Image quality = 4 in one of the contrast weightings led to exclusion of the evaluation procedure. For each patient, a data set of 108 contrast-weighted MR images (72 slices for the 3D TOF and 12 slices for T1w, T2w, and PDW) of the carotid arteries was obtained. The images were evaluated by two experienced reviewers. A consensus opinion was reached for each image analysis. The reviewers were blinded to the patient's clinical history at the time of image analysis. To determine the lesion type in accordance with the modified AHA criteria, the carotid atherosclerotic plaque in the 108 images of each patient was identified and 6 sequentially acquired slices of each contrast weighting in the region of the minimum lumen area were selected for evaluation. The carotid atherosclerotic plaque in these selected slides was ascribed to one of the six classification types according to the following modified AHA criteria: Type I-II shows near-normal wall thickness without calcification. Type III represents diffuse intimal thickening or small eccentric plaque without calcification. Type IV-V is characterized by a lipid or necrotic core surrounded by fibrous tissue with possible calcification. Type VI shows a complex plaque with possible surface defect, hemorrhage or thrombus. Type VII represents a calcified lesion. Type VIII is characterized by a fibrotic plaque without a lipid core and with possible small calcifications.

### Flow Cytometry

For analysis of circulating PCs, blood samples were taken at the date of the MRI examination and were processed immediately (<2 h). Mononuclear cells enriched from citrate phosphate dextrose acid-anticoagulated blood samples by Ficoll (Ficoll-Paque PLUS, Amersham Biosciences, Uppsala, Sweden) density gradient centrifugation were stained as previously described by our study group [34,35].

Vital CD133^+ ^PCs were determined using anti-CD133, anti-CD34, 7-AAD, and anti-CD45. CD133 and CD34 are surface markers of progenitor cells. The expression of CD34 and CD133 diminish with maturation and differentiation [36]. CD45 is present on all human leucocytes. On CD133+ PCs the expression of CD45 is low [37]. 7-AAD(7-amino-actinomycin D) was used for the exclusion of nonviable cells [38].

Vital VEGFR2^+^CD34^+ ^PCs were determined using anti-VEGFR2, anti-CD34, 7-AAD, and anti-CD45. VEGFR2 is a receptor of Vascular Endothelial Growth Factor(VEGF) that is expressed on endothelial cells [39]. As VEGFR2^+^CD34^+ ^PCs express endothelial markers and progenitor markers, they are classified as endothelial progenitor cells [40].

Surface expression of the SDF-1 receptor CXCR4 on CD133^+ ^PCs was analyzed using anti-CXCR4, anti-CD133, anti-CD45, and 7-AAD. In order to preserve CXCR4, heparin-blood was incubated with the described antibodies, Ficoll centrifugation was not performed. Fluorescence isotype-matched antibodies were used as controls. Flow cytometric analysis was performed using a FACS Calibur (BD Biosciences, Mountain View, CA, USA). Fluorescence intensity of at least 100 000 cells was recorded and analyzed using CellQuest software (BD Biosciences). Results of flow cytometry and MRI scans were analyzed by different investigators, each without knowledge of the other results.

Antibodies used were:

- PE-conjugated anti-CD133 (clone AC133) (Miltenyi Biotec, Auburn, USA)

- FITC-conjugated anti-CD34 (clone 8G12), 7-AAD and APC-conjugated anti-CD45 (clone 2D1) (Becton Dickinson, Heidelberg, Germany)

- PE-conjugated anti-VEGFR2 (clone 89106) and FITC-conjugated anti-CXCR4 (clone 12G5) (R&D Systems, Minneapolis, USA).

### Statistical Analysis

Differences between two groups were analyzed by Mann-Whitney *U*-test for interval variables and by chi-squared test for categorical variables (SPSS 16.0 for windows). A p-value < 0.05 was regarded as significant.

## Results

### Clinical and MRI Data

**Figure 1:**
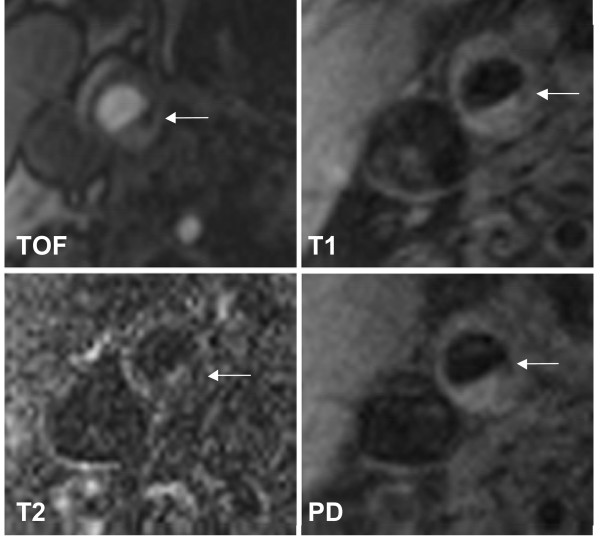
**Example of lesion type IV-V in the right internal carotid artery**. The necrotic core (←) shows low-signal intensity (SI) on both T1w and TOF images, but low SI to iso-SI on PDW and T2w images. Original magnification × 25.

**Figure 2:**
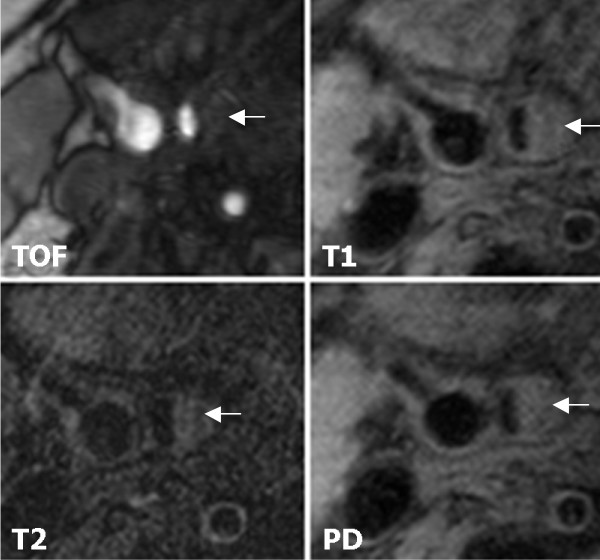
**Example of lesion type VIII in the right internal carotid artery**. Lesion type VIII (←) in the right internal carotid artery. MR images show iso- to slightly high-SI. Original magnification × 25.

We recruited 31 subjects with asymptomatic carotid artery stenosis. Twenty-three plaques were classified as stable according to the modified AHA-classification (types III, VII, and VIII) and 8 were classified as unstable (types IV-V and VI). Figure 1 shows a representative case of lesion type IV-V, Figure 2 shows a representative case of lesion type VIII. Subjects with stable plaques did not differ significantly from the subjects with unstable plaques concerning age, sex, and cardiovascular risk factors (Table 1).

**Table 1:** Baseline Characteristics and Lesion Type

	Subjects with Stable Plaques	Subjects with Unstable Plaques	
	(n = 23)	(n = 8)	p
Age, mean (range)	73 (61-84)	70 (59-84)	n.s.
Male, N (%)	17 (73.9%)	5 (62.5%)	n.s.
Hypertension, N (%)	21 (91.3%)	7 (87.5%)	n.s.
Atrial Fibrillation, N (%)	0	0	
Hyperlipidemia, N (%)	17 (73.9%)	7 (87.5%)	n.s.
Type II diabetes mellitus, N (%)	6 (26.1%)	2 (25%)	n.s.
Current or former smoker, N (%)	13 (56.5%)	5 (62.5%)	n.s.
Medication			
Statin, N (%)	19 (82.6%)	7 (87.5%)	n.s.
Thrombocyte aggregation inhibitors, N (%)	23 (100%)	8 (100%)	n.s.
Plaque type, N (%)			
III	2 (8.7%)		
IV/V		7 (87.5%)	
VI		1 (12.5%)	
VII	13 (56.5%)		
VIII	8 (34.8%)		

Data are shown as mean[median] ± sem

### Circulating CD133^+ ^Progenitor Cells and VEGFR2^+^CD34^+ ^Progenitor Cells

**Figure 3:**
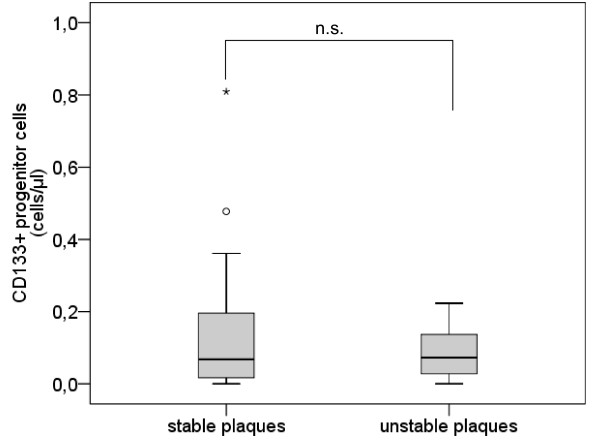
**Number of CD133^+ ^progenitor cells**. Boxplot demonstrating the number of CD133^+ ^progenitor cells (/μl) in subjects with stable plaques and subjects with unstable plaques. The top of the box represents the 75th percentile, the bottom of the box represents the 25th percentile, the center horizontal line represents the 50th percentile. The whiskers represent the highest and lowest values that are not outliers or extreme values. Outliers (between 1.5 and 3 times the interquartile range) are represented by circles and extreme values (more than 3 times the interquartile range) by asterisks.

The number of CD133^+ ^PCs did not differ between subjects with unstable plaques and subjects with stable plaques (mean [median] ± sem 0.14 [0.07] ± 0.04 cells/μl vs. 0.09 [0.07] ± 0.03 cells/μl, p = 0.982) (Table 2, Figure 3).

**Table 2:** Progenitor Cells in Subjects with Unstable Plaques and Subjects with Stable Plaques

	Subjects with Stable Plaques	Subjects with Unstable Plaques	
	(n = 23)	(n = 8)	p
CD133^+ ^progenitor cells (cells/μl)	0.14 [0.07] ± 0.04	0.09 [0.07] ± 0.03	n.s.
VEGFR2^+^CD34^+ ^progenitor cells (cells/μl)	0.15 [0.12] ± 0.04	0.17 [0.14] ± 0.04	n.s.
CXCR4 surface expression on CD133^+ ^progenitor cells	65.78 [62.6] ± 3.76	86.74 [87.57] ± 5.64	0.009

**Figure 4:**
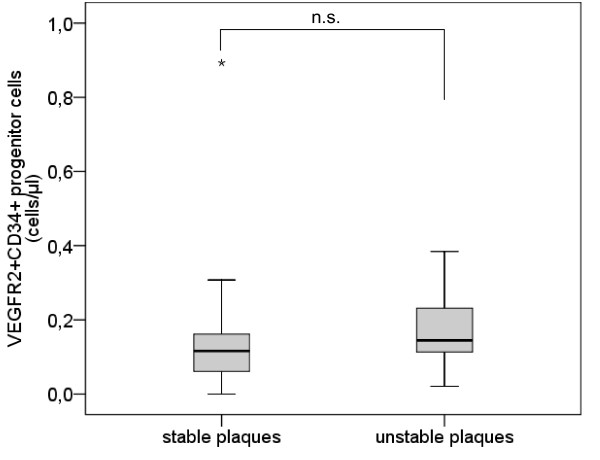
**Number of VEGFR2^+^CD34^+ ^progenitor cells**. Boxplot demonstrating the number of VEGFR2^+^CD34^+ ^progenitor cells (/μl) in subjects with stable plaques and subjects with unstable plaques. The top of the box represents the 75th percentile, the bottom of the box represents the 25th percentile, the center horizontal line represents the 50th percentile. The whiskers represent the highest and lowest values that are not outliers or extreme values. Outliers (between 1.5 and 3 times the interquartile range) are represented by circles and extreme values (more than 3 times the interquartile range) by asterisks.

The analysis of VEGFR2^+^CD34^+ ^PCs presents a similar pattern without significant difference between subjects with stable plaques and subjects with unstable plaques (mean [median] ± sem 0.15 [0.12] ± 0.04 cells/μl vs. 0.17 [0.14] ± 0.04 cells/μl, p = 0.219) (Table 2, Figure 4).

### Surface Expression of CXCR4 on CD133^+ ^Progenitor Cells

**Figure 5:**
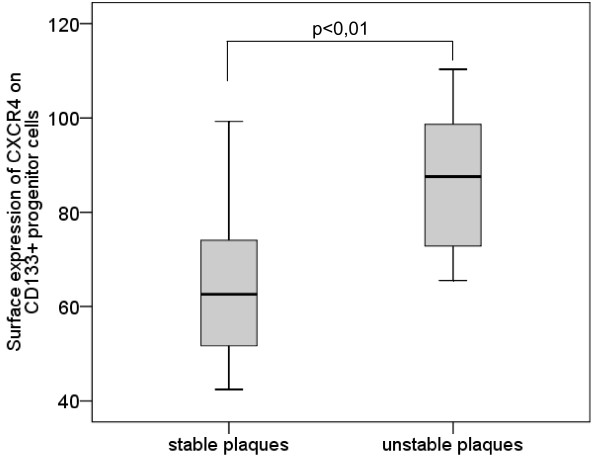
**Surface expression of CXCR4 on CD133^+ ^progenitor cells**. Boxplot demonstrating the surface expression of CXCR4 on CD133^+ ^progenitor cells in subjects with stable plaques and subjects with unstable plaques. The top of the box represents the 75th percentile, the bottom of the box represents the 25th percentile, the center horizontal line represents the 50th percentile. The whiskers represent the highest and lowest values.

The surface expression of CXCR4 on CD133^+ ^PCs was significantly higher in subjects with unstable plaques than in subjects with stable plaques (mean [median] ± sem 86.74 [87.57] ± 5.64 vs. 65.78 [62.6] ± 3.76, p = 0.009) (Table 2, Figure 5).

## Discussion

We have demonstrated that the number of CD133^+ ^PCs and VEGFR2^+^CD34^+ ^PCs does not differ significantly in subjects with stable carotid plaques compared with subjects with unstable carotid plaques. Yet surface expression of CXCR4 on circulating CD133^+ ^PCs was associated with plaque instability. Only a limited number of studies have analyzed the correlation between PCs and atherosclerotic lesions. Vasa et al. demonstrated that atherosclerotic risk factors inversely correlate with the number of circulating VEGFR2^+^CD34^+ ^PCs [25]. Consistently, subjects with the presence of carotid, aortic, or femoral plaques presented a decreased number of PCs. The presence of carotid plaques in particular entailed a reduction of 48% of VEGFR2^+^CD34^+ ^PCs compared with subjects without plaques [31]. It is therefore supposed that the number of circulating PCs is associated inversely with the degree of atherosclerotic disease. Although we can presume a decreased level of PCs in the subjects of our study because of their risk profile and the presence of plaques, we did not observe differences in the number of circulating PCs depending on plaque morphology. Thus there is no indication that different types of plaque influence the mobilization of PCs unequally. But it can not be ruled out that a higher consumption of PCs compensates a higher mobilization. So it also could be possible that the mobilization of PCs in subjects with unstable plaques and subjects with stable plaques differ even if the absolute number of circulating PCs is equal.

As the number of circulating PCs is low, not only their mobilization from the bone marrow but also their homing to the damaged tissue is of particular importance. The cytokine SDF-1 (stromal cell-derived factor 1) and its only known receptor CXCR4, which is expressed on PCs, seem to play an important part in the homing process [41,42]. Additionally, the CXCR4/SDF-1 interaction influences proliferation and mobilization of PCs from the bone marrow [43,44]. In addition to the number of circulating PCs, we therefore also analyzed the expression of their surface receptor CXCR4, in order to evaluate their homing process to the affected tissue. In contrast to other studies we did not evaluate the number of CXCR4^+ ^cells but the expression of CXCR4 on CD133^+ ^PCs as a functional parameter.

This is the first study to show an increased expression of CXCR4 on CD133^+ ^PCs in subjects with unstable plaques compared with subjects with stable plaques. Our results suggest that not the number of circulating PCs but their activation is associated with severity of atherosclerotic disease. As we are not able to detect the functional PCs in the injured tissue, analyzing CXCR4 on circulating PCs provides a promising auxiliary parameter.

Our results indicate that the recruitment of the PCs to the injured tissue in patients with unstable plaques may be elevated and the PCs can better become functional even if patients with unstable plaques present the same number of PCs as patients with stable plaques. There are two possible mechanisms of an increased recruitment of PCs to the plaque in patients with unstable plaques.

First, unstable plaques could activate the endogenous repair mechanism by recruiting PCs to the plaques. As PCs are involved in the reendothelialization of damaged endothelium, PCs are regarded as an endogenous repair mechanism [19]. Because of the enhanced presence of leukocytes in unstable plaques and their production of cytokines, CXCR4 can be upregulated and can therefore contribute to the recruitment of PCs to the plaque via the CXCR4/SDF-1 interaction. Thus the inflammatory process of an unstable plaque could trigger enhanced recruitment of PCs.

Another mechanism could be the potential unfavorable effect of PCs by induction of neoangiogenesis, thereby causing plaque instability. The role of PCs in neovascularization has been investigated by several groups [45-48]. In atherosclerotic lesions, neoangiogenesis could result in the entry of inflammatory cells and cytokines into the lesions and therefore promote the instability of plaques. Neovessel content was significantly increased in ruptured plaques compared with nonruptured plaques in the human aorta, so neovascularization could also contribute to plaque rupture [49,50]. Therefore, an enhanced recruitment of PCs to the plaques could aggravate plaque instability. However, the MRI-protocol used for our study did not allow to assess plaque neovascularisation. So we were not able to analyze a possible association between plaque neovascularisation and PCs.

It is also thinkable that the lower expression of CXCR4 in subjects with stable plaques suggests the occurrence of premature PCs caused by a higher consumption of PCs. The lower expression of CXCR4 on CD133^+ ^PCs could therefore be seen as a marker for a higher consumption of CD133^+ ^PCs meaning a raised endogenous repair mechanism.

As surface expression of CXCR4 on CD133^+ ^PCs is significantly higher in subjects with unstable plaques than in subjects with stable plaques, CXCR4 represents a promising candidate for a serological biomarker of plaque stability. Because of the low number of analyzed subjects, the results must be confirmed in a larger study.

## Conclusions

Our results demonstrate an association between circulating PCs and plaque stability in carotid artery stenosis. Subjects with unstable plaques showed a higher expression of the surface receptor CXCR4 on CD133^+ ^PCs than did subjects with stable plaques, suggesting differences in the recruitment of PCs to their target, even if the number of circulating PCs was equal. PCs may therefore play a role in the endogenous repair mechanism in atherosclerotic lesions. On the other hand, it must be kept in mind that PCs may also have a negative effect on plaque stability by stimulation of neovascularization.

## Competing interests

The authors declare that they have no competing interests.

## Authors' contributions

DS conceived and designed the research, acquired the data, analyzed and interpreted the data, performed statistical analysis and drafted the manuscript. LE conceived and designed the research, acquired the data, analyzed and interpreted the data, performed statistical analysis and drafted the manuscript. PZ acquired the data, analyzed and interpreted the data and made critical revision of the manuscript. IO conceived and designed the research, analyzed and interpreted the data and made critical revision of the manuscript. RF analyzed and interpreted the data and made critical revision of the manuscript. SS performed statistical analysis, analyzed and interpreted the data and made critical revision of the manuscript. BH analyzed and interpreted the data, handled funding and supervision and made critical revision of the manuscript. HP conceived and designed the research, analyzed and interpreted the data, handled funding and supervision and made critical revision of the manuscript. All authors read and approved the final manuscript.
